# Remote magnetic navigation vs. manual navigation for ablation of ventricular tachycardia: a meta-analysis

**DOI:** 10.1007/s12471-015-0734-1

**Published:** 2015-07-28

**Authors:** Y. Wu, K.-L. Li, J. Zheng, C.-Y. Zhang, X.-Y. Liu, Z.-M. Cui, Z.-M. Yu, R.-X. Wang, W. Wang

**Affiliations:** 1 Department of Cardiology, Wuxi People’s Hospital Affiliated to Nanjing Medical University, 214023 Wuxi, Jiangsu China; 2 Wuxi Center for Disease Control and Prevention, 214023 Wuxi, Jiangsu China

**Keywords:** Remote magnetic navigation, Manual catheter navigation, Ventricular tachycardia, Ablation

## Abstract

**Background:**

The purpose of this study was to prospectively evaluate the efficacy and safety of remote magnetic navigation (RMN) in comparison with manual catheter navigation (MCN) in performing ventricular tachycardia ablation.

**Methods:**

An electronic search was performed using PubMed (1948–2013) and EMBASE (1974–2013) studies comparing RMN with MCN which were published prior to 31 December 2013. Outcomes of interest were as follows: acute success, recurrence rate, complications, total procedure and fluoroscopic times. Standard mean difference (SMD) and its 95 % confidence interval (CI) were used for continuous outcomes; odds ratios (OR) were reported for dichotomous variables.

**Results:**

Four non-randomised studies, including a total of 328 patients, were identified. RMN was deployed in 191 patients. Acute success and long-term freedom from arrhythmias were not significantly different between the RMN and control groups (OR 1.845, 95 % CI 0.731–4.659, *p* = 0.195 and OR 0.676, 95 % CI 0.383–1.194, *p* = 0.177, respectively). RMN was associated with less peri-procedural complications (OR 0.279, 95 % CI 0.092–0.843, *p* = 0.024). Shorter procedural and fluoroscopy times were achieved (95 % CI -0.487 to -0.035, *p* = 0.024 and 95 % CI -1.467 to -0.984, *p*<0.001, respectively).

**Conclusion:**

The acute and long-term success rates for VT ablation are equal between RMN and MCN, whereas the RMN-guided procedure can be performed with a lower complication rate and less procedural and fluoroscopic times. More prospective randomised trials will be needed to better evaluate the superior role of RMN for catheter ablation of ventricular tachycardia.

## Introduction

Catheter ablation was introduced into clinical electrophysiology in the 1980s [1]. Considering numerous secondary effects and the low efficacy of antiarrhythmic drugs, catheter ablation is now a well-established therapy of choice for many types of arrhythmias, including ventricular tachycardia (VT) [2]. However, catheter ablation is a demanding procedure not only because it relies on an experienced team of electrophysiologists but also because it requires a high level of radiation exposure. Until now, all of the above-mentioned techniques are still based on manual catheter navigation (MCN) within patient hearts. Further technological developments such as electroanatomical mapping, integration of cardiac imaging, and improved catheter design have been implemented to improve the consistency of procedural outcomes [3, 4]. The innovation of remote magnetic navigation (RMN) has offered important theoretical advantages in safety due to the atraumatic catheter design, less physical stress and less radiation exposure for physicians [4, 5].

Since the first published report of RMN used in humans in 2003 [6], numerous investigators have already examined the safety and feasibility of the RMN system for ablation of a variety of tachyarrhythmias [7, 8]. However, the data obtained from the existing literature regarding the incremental efficacy and safety of this system are mostly limited to small trials and case series. The acceptability of RMN as an alternative tool for the ablation of VT remains uncertain. The objective of the present study was to evaluate the safety and long-term efficacy of RMN as compared with MCN for VT ablation.

## Methods

### Search strategy and eligibility of relevant studies

All the case-control studies were identified by searching electronic databases (PubMed from 1948 to 2013 and EMBASE from 1974 to 2013), using the following key words: ‘ventricular tachycardia’, ‘ablation’ and ‘remote magnetic navigation’. We also searched the Cochrane and DARE databases for additional records. The search was limited to English language papers and studies in humans. The search was performed on 31 December 2013. If there was more than one study with the same population by different investigators or overlapping data by the same authors, we selected the most recent or complete articles with the largest number of subjects. Studies included in our meta-analysis had to meet the following criteria: (1) inclusion of patients with VT (either paroxysmal or persistent); (2) comparison between VT ablation obtained with the RMN and MCN control system; and (3) report of results of at least one relevant outcome, including acute success rate in eliminating VT, procedure complications, recurrence rate, and procedural data (e.g. total duration of the procedure, fluoroscopic time). Those studies without controls and duplicates of previous publication were excluded.

### Study selection

The literature search was conducted by one investigator (Y.W.). Two researchers (Y.W. and W.W.) independently selected studies for inclusion according to eligibility criteria. Disagreements between reviewers were resolved by consensus; if no agreement could be reached, it was agreed that a third author (RX.W.) would decide.

### Data extraction

Two investigators independently extracted the data and reached consensus on all items. In the present study, the following information was extracted from each publication: (1) the first author’s last name, year of publication, characteristics of trial participants (age, sex, follow-up time and country where the study was performed); (2) primary outcomes: acute success rate in eliminating VT and recurrence rate at the end of the follow-up (independently from the length and intensity of the follow-up in each study); (3) secondary outcomes: procedural complications, total procedure time and fluoroscopic times.

### Statistical analysis

Odds ratio (OR) was the primary measure of treatment and side effects; the 95 % confidence interval (CI) for OR was calculated. The statistical significance of the pooled OR was determined with the Z-test. Standard mean difference (SMD) and its 95 % CI were used for continuous outcomes. The statistical heterogeneity among studies was assessed with the χ^2^ based Q-test [9], and a *p*-value of < 0.10 was considered significant, the summary OR estimate of each study was calculated by the random-effects model (the DerSimonian and Laird method) [10], otherwise, fixed-effects model (the Mantel-Haenszel method) was used [11]. We also used metareg analysis to assess the source of heterogeneity. To test the publication bias, funnel plots and Egger’s linear regression test was applied [12]. All analyses were done with Stata software (version 10.0; StataCorp LP, College Station, TX), using two-sided p-values: *p* < 0.05 was considered significant.

## Results

### Study selection

**Fig. 1:**
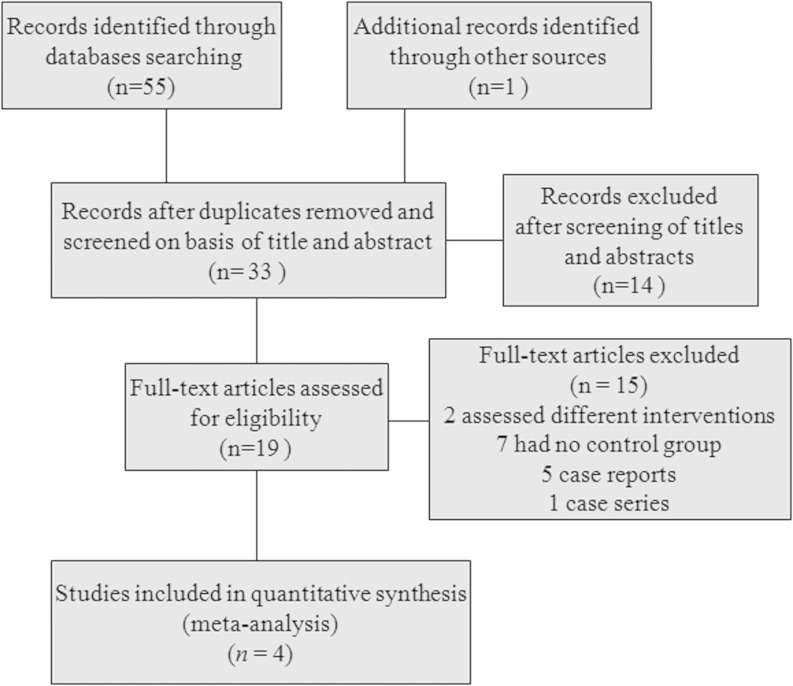
The flow diagram of studies selection

There were 56 published articles relevant to the search words. Figure 1 shows the process of inclusion and exclusion of associated studies. Exclusion of duplicate references left 33 records. Of these, 14 studies were discarded because after reviewing the abstracts it appeared that these papers clearly did not meet the eligibility criteria. The full text of the remaining 19 citations was examined in detail. It then appeared that a further 15 studies did not meet the inclusion criteria: 2 studies considered different interventions, 7 studies did not have a control group, 5 were case reports and 1 a case series. Finally, four trials were included in our analysis [13–16].

### Characteristics of the studies

Four retrospective studies comparing RMN and MCN were identified, and the characteristics of eligible studies are summarised in Table 1. Overall, 328 participants were considered, with the number of participants ranging from 30 to 113. Two studies were published in 2012 [13, 14], another two trials were published in 2011 [15] and 2013 respectively [16]. Clinical follow-up periods varied from 13 to 22 months. Follow-up frequency differed among these studies.

**Table 1:** Characteristics of studies selected in the meta-analysis

Study	Year	Country	Study design	Number of paticipants	Mean age (year)	Male	Follow-up length (months)	Ref.
Dinov et al.	2012 [13]	Germany	Pubhshed NRS	102	RMN:69 ± 10 MCN:66 ± 9	88/102	RMN:13.0 ± 9.0 MCN:14.0 ± 9.8	13
Szili-Torok et al.	2012 [14]	the Netherlands	Pubhshed NRS	113	RMN:51 ± 15 MCN:49 ± 17	82/113	RMN:20 ± 11 MCN:20 ± 10	14
Bauernfeind et al.	2011 [15]	the Netherlands	Pubhshed NRS	83	–	–	–	15
Zhang et al.	2013 [16]	China	Pubhshed NRS	30	RMN:41.7 ± 9.1 MCN:46.5 ± 7.4	8/30	22.1 ± 4.6	16

*NRS* non-randomised controlled study, *RMN* remote magnetic navigation, *MCN* manual catheter navigation.

### Acute success rate

**Fig. 2:**
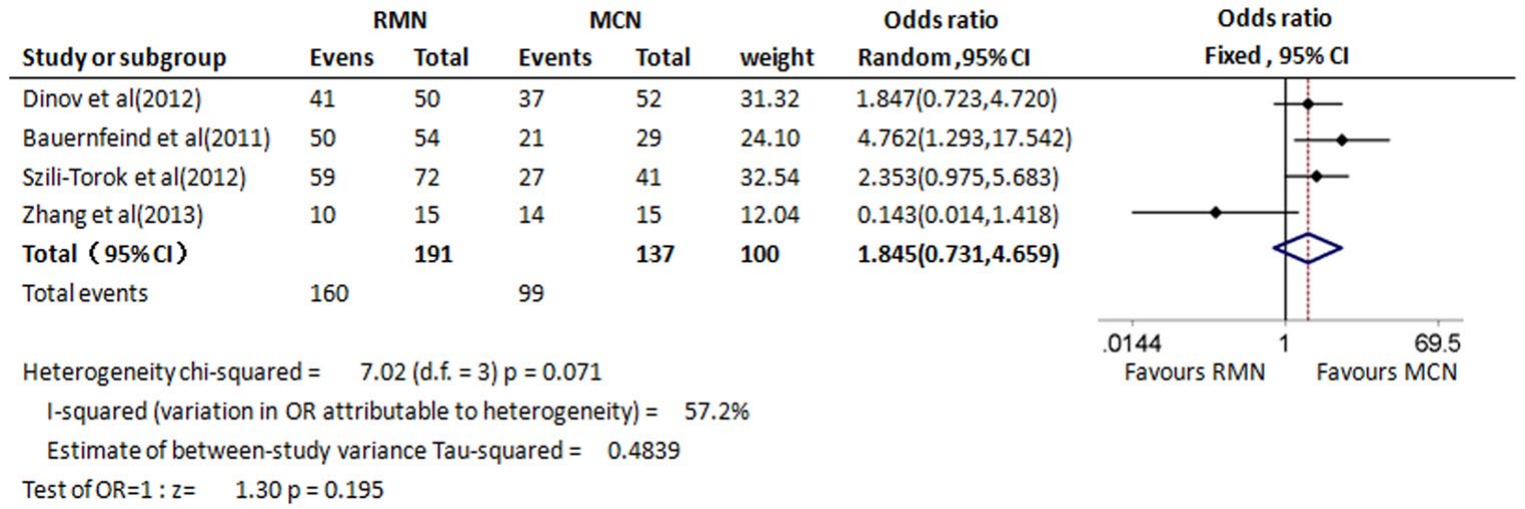
Forest plot of acute success rate between RMN procedure and MCN procedure. The squares and horizontal lines correspond to the study-specific odds ratio (OR) and 95 % confidence interval (CI). The area of the squares reflects the study-specific weight (inverse of the variance). The diamond represents the pooled OR and 95 % CI

Data on acute success rates were all reported and assessed in 328 participants of all these four studies [13–16]. Acute success rate was not statistically significant between the RMN and MCN procedure (OR 1.845, 95 % CI 0.731–4.659, *p* = 0.195). Substantial heterogeneity was present in the comparison (*I* ^*2*^ = 57.2 %, *p* = 0.071; Fig. 2).

### Recurrence rate

**Fig. 3:**
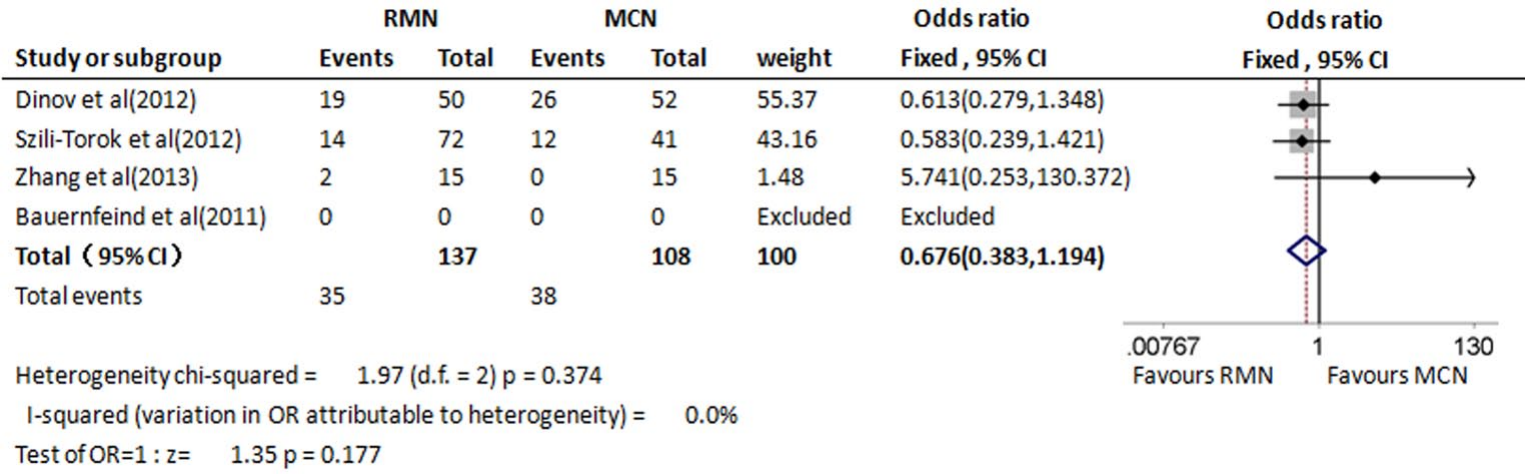
Forest plot of recurrence rates of VT in the RMN and MCN group

The proportion of patients with recurrences of VT or other ventricular tachyarrhythmias was evaluated at the end of the follow-up periods in three trials, including 245 patients [13, 14, 16]. The follow-up in the studies lasted from 13 to 22 months, and the trials were heterogeneous for the intensity and the follow-up methods. The recurrence rates of VT and other ventricular tachyarrhythmias were not statistically superior in the RMN group than the MCN group (OR 0.676, 95 % CI 0.383–1.194, *p* = 0.177; Fig. 3).

### Complications

**Fig. 4:**
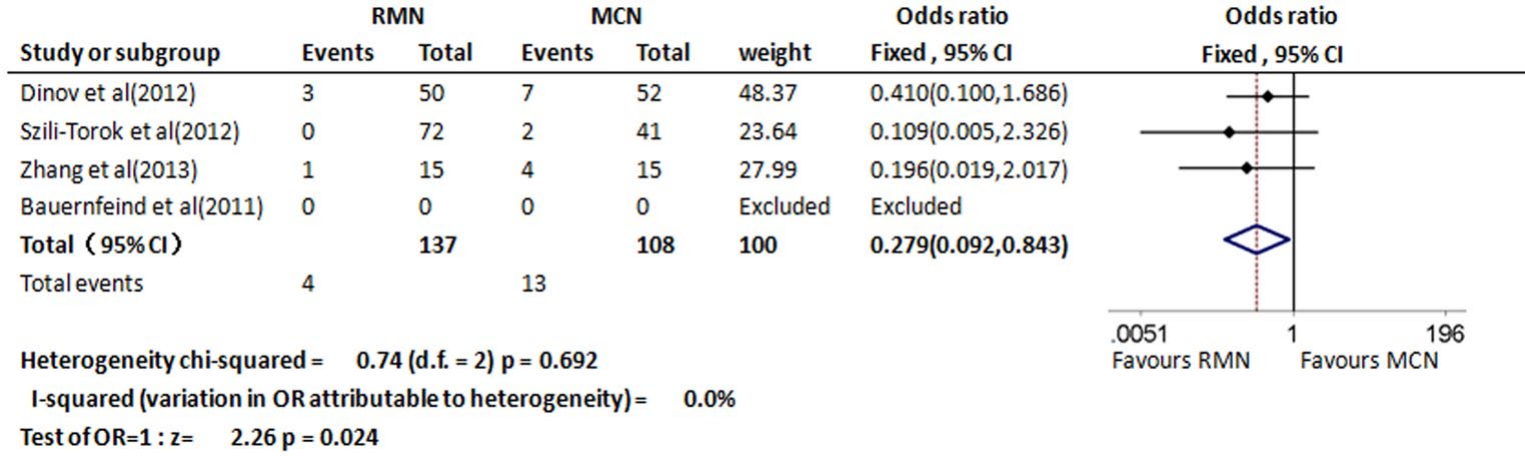
Forest plot of procedure complications between the RMN and MCN group

The data on procedure complications were reported in these three studies including 245 patients [13, 14, 16]. Major complications such as death, cardiac perforation, cardiac tamponade, pseudoaneurysm, arteriovenous fistula, pericardial effusion, and right bundle branch block, were rare events and occurred in 4 of 137 patients in the RMN group (2.9 %) and in 13 of 108 (12.0 %) in the MCN group. The difference between the two groups was statistically significant (OR 0.279, 95 % CI 0.092–0.843, *p* = 0.024; Fig. 4).

### Procedural time and fluoroscopic time

All four studies reported data on total procedural time and fluoroscopic time [13–16]. Overall, the ablation procedures were significantly shorter and fluoroscopic time was significantly reduced in the RMN group (95 % CI − 0.487 to − 0.035, *p* = 0.024 and 95  % CI − 1.467 to − 0.984, *p* < 0.001, respectively). A substantial heterogeneity was found for procedural time (*I* ^*2*^ = 85.1 % and *p* < 0.001).

### Test of heterogeneity

There was a significant result for test of heterogeneity by acute success rate (*P*_*heterogeneity*_ = 0.071, *I* ^*2*^ *=* 57.2 %) and procedural times (*P*_*heterogeneity*_ < 0.001, *I* ^*2*^ *=* 85.1 %). In order to detect the source of this heterogeneity, we performed metareg analysis using three factors of the included studies, i.e. published year, country of study and number of participants. However, none of the three factors were found to be the source of heterogeneity (total Tau-squared = 0.4839, while application of none of the three factors in metareg analysis can reduce this value) in the analysis of acute success. The same method was also used to detect the source of heterogeneity in the analysis of procedural times, and three factors were not contributed to the source of heterogeneity. Therefore, we speculated that the identified heterogeneity may be attributed to some other unknown factors, which was inconsistent among all the included studies.

### Publication bias

Begg’s funnel plot and Egger’s test were performed to assess the publication bias of the currently available literature. As a result, the shape of the funnel plots reveals evidence for obvious asymmetry in all the comparison models. Then, the Egger’s test was used to provide statistical evidence for funnel plot symmetry. The results also show the evidence of publication bias (*p* < 0.001).

## Discussion

Catheter-based procedures have become a mainstay for the treatment of cardiac arrhythmias, with high success rates, low complications, and improved quality of life. Manual navigation of the ablation catheter is often challenging and associated with a long procedure time and excessive X-ray exposure, especially for ablation of complex arrhythmias. The optimal technology in future is therefore in favour of an approach that is at least as effective as the MCN technique but has an improved safety profile regarding potential complications and other variables, such as X-ray exposure and time for patients and operators. The increased use of RMN technology has occurred primarily because of potential unique capabilities and benefits such as increased precision with catheter movement and control, improved catheter stability with constant tissue contact, decreased risk of cardiac perforation due to the compliant nature of the catheter, and decreased fluoroscopic exposure for both patients and physicians [17]. How and if these potential benefits translate into clinical outcomes remain to be determined. The current guidelines recognise the lack of sufficient data on the efficacy and safety of this technology in the field of catheter ablation of VT [18]. Our meta-analysis showed similar efficacy outcomes (acute success rate and recurrence rate) with the use of RMN when compared with MCN for VT ablation. RMN was superior in safety as compared with manual navigation resulting in a lower number of complications as well as lower procedural and fluoroscopic times. Therefore, the use of RMN in VT ablation in centres where RMN can be available should be a reasonable alternative based on the results.

While the procedure for VT ablation performed with RMN is not statistically superior to the MCN procedure in achieving acute and chronic success rates, in this meta-analysis, a statistically significant reduction in the incidence of major complications was noted with RMN. Manual navigation of catheters in the human heart has limitations as follows: some regions are difficult to reach, and compromised catheter positioning may result in insufficient lesion formation [19, 20]. Catheter movement in some positions is accompanied by the risk of major complications, including pericardial effusion or cardiac tamponade [20]. Although several pre-defined catheter curves were introduced to help appropriate lesion delivery, there are no optimal curves available for the treatment of paediatric patients with small hearts, patients with complex congenital heart defects, or some types of VTs [21]. The major advantage of the remote magnetic navigation system is its ‘floppy’ ablation catheter. Because of this floppiness, there is an enormous freedom of movement of the ablation catheter. The operator can easily reach any desirable site on the endocardium or epicardium due to the absence of a predefined curve. As the atraumatic catheter design is less harmful to the cardiac wall, this ablation technology can also be safely used by less experienced operators [22].

In this meta-analysis, both procedural and fluoroscopic times were reduced in RMN as compared with the conventional groups. The value of assessing procedural time as a benefit for RMN may be limited. Many studies were performed in the early experience with RMN, the procedural time has since been improved with increasing experience of RMN technology. A learning curve has been reported for operators as well as lab staff for system and patient preparation and setup. However, even with increasing experience with the technology, procedure time is unlikely to be the area where RMN has the most benefit for patients and physicians. The reduction in radiation exposure is the most important advantage of remote magnetic catheter navigation, as there is a risk for radiation-induced diseases. The importance of reduction in radiation exposure from fluoroscopy in young adults was well recognised by Roudijk et al. [23]. The high manoeuvrability and atraumatic design of the RMN-guided ablation catheter allows navigation without constant fluoroscopic control, while re-imaging is typically required after each repositioning of the manual-guided catheter. Therefore, theoretically, RMN can decrease the radiation exposure both to operators and patients. Furthermore, remote navigation removes the operator’s need to be at the bedside for catheter manipulation and in early studies was shown to decrease acute and chronic fluoroscopic exposure [24].

Some limitations of the meta-analysis should be mentioned. Firstly, lack of availability of the original data from the reviewed studies limited our further evaluation of the source of heterogeneity, and the small number of studies that met the requirements may cause the publication bias. As often happens for medical devices, we retrieved only a limited number of low-quality studies, all non-randomised controlled studies (NRSs). Although the results from NRSs are generally accepted as basis for taking clinical decisions, they are inherently susceptible to bias, with retrospective ones at the lower rank in the hierarchy of evidence. Another limitation of our review is that the external validity of the results is uncertain. Different levels of experience among operators, incomplete data for many study outcomes, single-centre based studies and the small sample size of both RMN group and MCN group limited the conclusions. Finally, as the trials included in the meta-analysis were not conducted in patients with right or left ventricular outflow tract arrhythmias, with or without structural heart disease, subgroup analysis cannot be performed, so the results cannot be applied in a particular clinical condition.

## Conclusion

VT ablation with RMN is equal to MCN in terms of acute and long-term success rates, whereas RMN-guided procedures can be performed with a lower complication rate and less procedural and fluoroscopic times. Heterogeneity was identified in the analysis of acute success and procedural time, and we performed metareg analysis using three factors of the included studies. However, none of the three factors were found to be the source of heterogeneity. We speculated that the identified heterogeneity might be attributed to some other unknown factors, which was inconsistent among all the included studies. Since our results are limited to existing studies which are small and not randomised, reaching definitive recommendations about the net benefit of RMN requires more collaborations among cardiac electrophysiology centres to implement randomised controlled trials enrolling a large number of patients. Prospective randomised trials following a rigorous pre-defined protocol will be needed to better evaluate the superior role of RMN for catheter ablation of VT.

### Conflict of interest

None declared.
